# Absence of population structure across elevational gradients despite large phenotypic variation in mountain chickadees (*Poecile gambeli*)

**DOI:** 10.1098/rsos.170057

**Published:** 2017-03-15

**Authors:** Carrie L. Branch, Joshua P. Jahner, Dovid Y. Kozlovsky, Thomas L. Parchman, Vladimir V. Pravosudov

**Affiliations:** Department of Biology and Graduate Program in Ecology, Evolution, and Conservation Biology, University of Nevada, Reno, Max Fleischmann Agriculture Building, 1664 N. Virginia Street, Reno, NV 89557, USA

**Keywords:** elevation gradient, mountain chickadee, genetic structure, local adaptation

## Abstract

Montane habitats are characterized by predictably rapid heterogeneity along elevational gradients and are useful for investigating the consequences of environmental heterogeneity for local adaptation and population genetic structure. Food-caching mountain chickadees inhabit a continuous elevation gradient in the Sierra Nevada, and birds living at harsher, high elevations have better spatial memory ability and exhibit differences in male song structure and female mate preference compared to birds inhabiting milder, low elevations. While high elevation birds breed, on average, two weeks later than low elevation birds, the extent of gene flow between elevations is unknown. Despite phenotypic variation and indirect evidence for local adaptation, population genetic analyses based on 18 073 single nucleotide polymorphisms across three transects of high and low elevation populations provided no evidence for genetic differentiation. Analyses based on individual genotypes revealed no patterns of clustering, pairwise estimates of genetic differentiation (*F*_ST_, Nei's D) were very low, and AMOVA revealed no evidence for genetic variation structured by transect or by low and high elevation sites within transects. In addition, we found no consistent evidence for strong parallel allele frequency divergence between low and high elevation sites within the three transects. Large elevation-related phenotypic variation may be maintained by strong selection despite gene flow and future work should focus on the mechanisms underlying such variation.

## 1. Introduction

Multiple evolutionary and environmental factors influence variation in local adaptation and population genetic structure across the landscape. Geographical isolation can lead to spatial genetic structure by directly limiting gene flow and allowing allele frequencies to differ as a result of genetic drift (isolation by distance [1]). In addition, ecological isolation of populations experiencing divergent selection can indirectly reduce gene flow as a result of selection against immigrants or by individual preferences to remain in different habitats [2–4]. Thus, the extent to which geography and local adaptation shape patterns of landscape genetic structure is jointly influenced by the strength of geographically based divergent selection and by geographical distribution, dispersal, and life history [5,6]. In addition, when adaptation occurs in the face of gene flow, genetic differentiation can be heterogeneous across the genome, with genetic regions involved in local adaptation restricted to small portions of the genome [7–9]. As a result, extensive genomic sampling may be required to detect fine-scale geographical genetic structure arising either directly or indirectly from spatially variable selection.

Spatially variable environments play a major role in ecological and evolutionary processes, and offer the opportunity for understanding the factors that shape phenotypic and population genetic variation. Habitats exhibiting environmental variation on relatively small spatial scales could improve our understanding of the relationship among environmental variation, local adaptation and population genetic structure. Montane habitats present a particularly useful setting to investigate these issues, because heterogeneity often occurs rapidly and predictably along elevation gradients [10,11]. Montane heterogeneity is manifested through predictable climatic and environmental variation, often resulting in variation between populations inhabiting different elevations. Elevation-related variation may be exhibited through differences in morphology, physiology, development and behaviour [11–25]. In some cases, elevation associated phenotypic differentiation coincides with population genetic structure [11,12,14,16,18,20,21,26,27], illustrating how divergent selection can shape phenotypic variation across small spatial scales when sharp ecological gradients reduce gene flow and lead to isolation by environment [5].

Mountain chickadees (*Poecile gambeli*) are resident, food-caching passerines that inhabit a range of elevations along the Western montane regions of North America [28]. These birds are scatter hoarders, meaning they cache (store) food items in multiple locations when food is abundant (summer/autumn) to use later when food is scarce (winter), and use spatial memory, at least in part, to relocate these caches [29]. Birds inhabiting higher elevations experience harsher winter conditions (lower temperature, more snow, extended periods of snow cover [30]) and probably have a higher reliance on previously cached food to survive winter compared to birds inhabiting lower, milder elevations [19].

Mountain chickadees, like other parids, remain in flocks of unrelated individuals throughout the winter and experience approximately 50% winter mortality [28]. As their winter survival is affected by the local environmental conditions, phenotypes enhancing winter survival would probably be favoured by selection (e.g. enhanced spatial memory for cache recovery). Indeed, high elevation birds (approx. 2400 m) cache more food items (3–4 times more than low elevation birds) [19,31] and exhibit superior spatial memory ability (high elevation birds perform twice as well on spatial memory tasks) [19] associated with significant differences in morphology of the hippocampus (high elevation birds have up to 20% larger hippocampal volume and up to 50% more hippocampal neurons) [19,32], the brain region underlying memory abilities, compared to low elevation birds (approx. 1900 m) [19,31,32]. While there are no direct estimates of heritability for such cognitive phenotypes, work in mammals suggests that spatial memory (*h*^2^ = 0.32–0.54) [33–36] and hippocampal volume (*h*^2^ = 0.29–0.95) [37,38] are moderately to highly heritable. In addition, the elevation-related differences we have documented in mountain chickadees have been found in juvenile birds prior to their first winter and, therefore, prior to the largest climate-related mortality event, further suggesting that differences found in spatial ability and hippocampus morphology may be heritable and capable of responding to selection [32].

In addition to elevation-related variation in spatial memory and neural morphology, these same populations of high and low elevation birds exhibit differences in social dominance. Low elevation males were dominant to high elevation males in pairwise interactions [39]. Differences in social dominance are particularly interesting given that dominant individuals tend to experience higher fitness (e.g. better access to food resources, territories and mates) compared to their subordinate counterparts (e.g. [40]). Taken together, existing evidence suggests that there are fitness consequences of moving between elevations. High elevation birds may have lower fitness at low elevations, owing to their socially subordinate status, while low elevation birds may have lower fitness at high elevations owing to their lower food caching propensity and inferior memory ability for retrieving caches, which may be required to survive in harsher high elevation environment. Furthermore, female mate choice could limit gene flow between high and low elevation populations. High elevation females prefer high elevation males over low elevation males in pairwise choice trials [22], and potentially use differences in song structure to discriminate males from high versus low elevations [23]. Finally, on average, low elevation birds begin breeding two weeks earlier than high elevation birds [41], providing yet another potential premating isolation mechanism.

Highly vagile organisms, such as birds, are often expected to show limited population structure, even over large geographical regions. Indeed, some formally recognized and phenotypically differentiated bird subspecies often show little or no evidence of genome-wide differentiation despite marked phenotypic differentiation [9,42,43]. Previous studies of mtDNA variation in mountain chickadees have indicated genetic structure of populations at broad geographical scales corresponding to eastern (Rocky Mountain) and western (Sierra Nevada and Cascades) clades but revealed little evidence for genetic structure across smaller geographical scales within these clades [44]. This is perhaps not surprising, as mountain chickadees are continuously distributed across the coniferous forests of the Sierra Nevada. However chickadees and tits from the genus *Poecile* are well known to be highly sedentary with rather short post-natal dispersal distances and limited to no movements once juveniles settle (e.g. [28,45]). The previous study of genetic variation in mountain chickadees sampled small numbers of molecular markers with limited resolution for understanding fine-scale population structure [44]. Indeed, some recent studies have documented fine-scale genetic structure within other avian populations across small geographical regions, which appear to be driven by strong ecological variation [20,21,46,47].

Recent innovations in DNA sequencing technology have drastically improved our ability to quantify fine-scale population genetic variation in ecologically significant but genomically understudied organisms [48–51]. Population genomic approaches can query thousands of sites across the genomes of many individuals and may reveal previously unrecognized genetic structure across environmental gradients over fine geographical scales [52,53]. Despite the accumulation of evidence indirectly suggesting that high and low elevation mountain chickadees may be locally adapted to their respective elevations (e.g. [54]), it remains unknown whether or not local adaptation and selection against migrants has generated ecological isolation across the elevation gradient. Here we use a genotyping-by-sequencing approach [49,55–57] to generate thousands of single nucleotide polymorphisms (SNPs) to assess genetic differentiation and population structure across six sampling sites; birds were sampled at high and low elevation across three transects. Our goal was to assess if high and low elevation birds exhibit fine-scale genetic structure that could potentially arise from divergent selection between high and low elevation habitats.

## 2. Methods

### 2.1. Tissue sample collection

**Figure 1.**
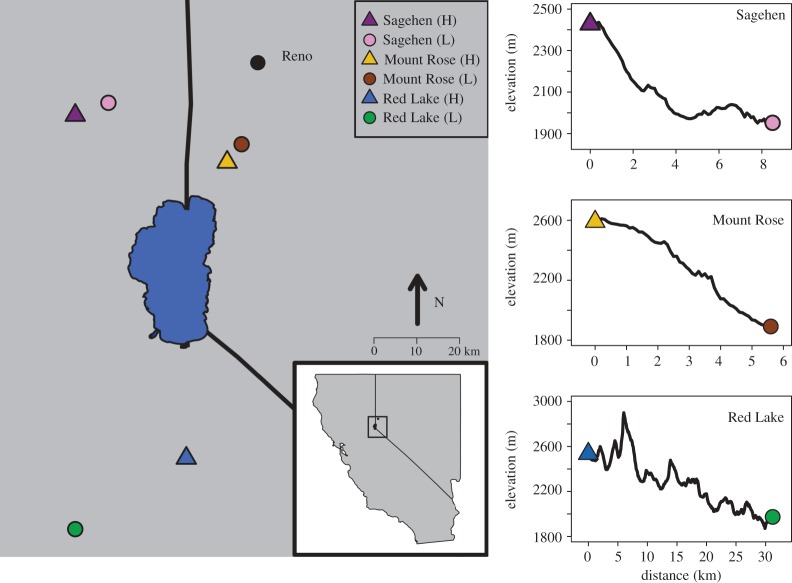
Map and elevation profiles of the six sites where mountain chickadees were sampled (created using R 3.3.0, R Core Team 2015). Inset shows region of California and Nevada where sites were sampled. Large blue shape in the middle of the inset represents Lake Tahoe. Panels show the elevation profiles found between the three pairs of elevational transects (points were sampled every 30 m as the duck flies between pairs of sites in ArcGIS). Triangles represent high elevation locations and circles represent low elevation locations.

In the autumn of 2013, we sampled 167 birds from three pairs of high (2535–2590 m) and low elevation sites (1891–2122 m) in the Sierra Nevada (table 1); Sagehen Experimental Forest, CA (exact same locations sampled as those used in all phenotypic variation studies [19,22,23,31,32,39]), Mount Rose, NV (exact same locations sampled as those used to show male song structure variation [23]), and Red Lake Peak, CA (no phenotypic variation data have been collected from these locations) (figure 1). Birds were captured using mistnets at established feeders at Sagehen Experimental Forest; the other locations do not have a feeder system in place. At Mount Rose and Red Lake Peak, a mistnet was set and mountain chickadee song was broadcast from the centre of the net using a FOXPRO Fury© playback speaker. Upon capture, approximately 100 µl of blood was collected from the basilic vein with a capillary tube and stored directly in Queen's lysis buffer (1 mM NaCl, 1 mM EDTA, 1 mM Tris).

**Table 1.** Sample sizes (*N*), latitude and longitude coordinates, and elevation (m) for each of the six locations where blood samples were collected for genomic analyses. (High (H) and low (L) elevation localities are delineated parenthetically.)

location	latitude	longitude	elevation (m)
Sagehen (H)	39° 25^′^ 7.73^″^	120° 18^′^ 24.72^″^	2428
Sagehen (L)	39° 26^′^ 42.36^″^	120° 13^′^ 02.01^″^	1952
Mount Rose (H)	39° 19^′^ 11.72^″^	119° 53^′^ 47.57^″^	2590.8
Mount Rose (L)	39° 21^′^ 27.99^″^	119° 51^′^ 26.95^″^	1891.59
Red Lake (H)	38° 41^′^ 47.09^″^	120° 00^′^ 25.35^″^	2535.33
Red Lake (L)	38° 32^′^48.59^″^	120° 18^′^24.97^″^	1973.58

### 2.2. Population genetic analyses

DNA was extracted from blood samples using a Qiagen DNeasy Blood and Tissue kit (Qiagen Inc., Valencia, CA, USA) and quantified using spectrophotometry on a QIAxpert machine (Qiagen, Inc). Reduced representation libraries for Illumina sequencing were constructed using a genotyping-by-sequencing (GBS) approach used in multiple previous studies [49,55–57]. Specifically, DNA fragments were cut using two restriction enzymes, EcoRI and MseI, which have cut sites evenly distributed throughout avian genomes [58]. Barcoded adaptors were ligated to the EcoRI cut site, which included an Illumina adaptor, an 8–10 bp barcode unique to each individual bird, and the bases matching the cut site. An MseI adaptor consisting of the opposite Illumina adaptor was ligated to the opposite ends (MseI cutsites) of the fragments. DNA from all individual reactions were pooled and PCR-amplified using Illumina primers. Finally, fragments between 350 and 450 bases in length were size selected using a BluePippin quantitative electrophoresis unit (Sage Science, Beverly, MA, USA). Single end, 100 base reads were generated on one lane of an Illumina HiSeq 2500 at the University of Texas Genomic Sequencing and Analysis Facility (UTGSAF, Austin, TX, USA).

The 100 bp reads generated on the HiSeq run were first filtered to remove contaminant DNA (e.g. *Escherichia coli*; PhiX) and low quality reads. A perl script was then used to identify individual barcodes, correct barcodes with errors, and remove reads containing sequences associated with Illumina adaptors or PCR primers. After this step, fragments were 86–88 bases in length. A random subset of 25 million reads was assembled *de novo* using the SeqMan ngen software (DNASTAR Inc.), specifying a minimum match percentage of 95 and a gap penalty of 30 (full details of parameter settings are available from the authors by request). Contigs were removed from the reference if they contained fewer than 10 reads, were over-assembled, or were not 84–90 bp in length. This step produced a reference of genomic regions sampled with our GBS approach, providing a template for subsequent reference guided assembly. DNA sequences from each chickadee were subsequently aligned to the reference with bwa v7.5 [59]using the aln and samse algorithms and an edit distance of 4. Because all sampled genomic regions begin with the EcoRI cut site and all HiSeq reads contained 100 bases of sequence, these alignments produced consistently rectangular contigs with even positional coverage.

Variant sites (i.e. SNPs) were called and quantified using samtools v.0.1.19 and bcftools v.0.1.19 [59,60]. SNPs were considered if at least 90% of individual birds had at least one read at the position, the site was biallelic, and the minor allele frequency was greater than 5%. For reference contigs containing multiple SNPs, a single SNP was randomly selected to increase independence of SNPs and to decrease the effect of linkage disequilibrium on subsequent analyses. For each bird, genotype likelihoods were calculated for each SNP using bcftools. Genotype likelihoods were initially stored in Variant Call Format (.vcf) and then converted to a composite genotype likelihood format. Genotype likelihood matrices and assembly related files are available at Dryad and additional information regarding parameter settings is available from the authors upon request.

To account for uncertainty associated with variation in coverage depth, a hierarchical Bayesian model [55] was employed to estimate genotype probabilities based on the genotype likelihoods estimated above. This model treats population allele frequencies as priors and simultaneously estimates both allele frequencies and genotype probabilities after accounting for variation in coverage. Essentially, individuals with low coverage for a given locus will have genotype probabilities more heavily informed by the prior (i.e. the allele frequency), while high coverage loci will have genotype probabilities with higher certainty of the homozygous or heterozygous genotypes. The model was run for 10 000 Markov chain Monte Carlo steps (thinning every other step) with a 6000 step burn-in to obtain the posterior estimates of genotype probabilities, which were subsequently stored as convenient composite genotype values (ranging from 0 to 2; 0 and 2 for homozygous and 1 for heterozygous genotypes) for use in all downstream analyses.

The distribution of genetic variation among individuals was first summarized with principal component analysis (PCA) using the *prcomp* function in R [57]. As a complementary approach to PCA, we performed discriminant analysis of principle components (DAPC) [61] using the adegenet package [62,63] in R. While PCA is inherently constrained to maximize the total variance explained in the data, DAPC maximizes the proportion of variance explained among groups of individuals [64]. Additionally, DAPC is useful for GBS datasets because it finds the most likely number of clusters within a dataset using k-means clustering and calculates individual assignment probabilities much faster than more computationally intensive Bayesian clustering algorithms commonly used in molecular ecology studies (e.g. Structure [65]). The most likely number of clusters for DAPC was calculated using the *find.clusters* function and the appropriate number of retained PCs was estimated using the *optim.a.score* function. For both PCA and DAPC, permutational multivariate analysis of variance (PERMANOVA [66]) implemented in the vegan package [67] of R was used as a post hoc test for genetic differentiation among sampling sites based on Euclidean distances of the first two ordination axes.

We summarized the degree of genetic differentiation among sampling sites by calculating Nei's genetic distance (*D* [68]) for each pairwise combination of sites. We also calculated pairwise genome-wide *F*_ST_ using Hudson's estimator [69] as an additional metric of differentiation among sites, and tested the significance of these estimates using a permutation-based approach. To test for the effects of geographical distance and elevational distance on genetic distances among sampling sites, we used a multiple regression on distance matrices (MRM [70]) using the ecodist package [71] in R, with pairwise genetic distance (*D*) as the response variable and pairwise elevational and geographical distance as the predictor variables. We estimated geographical distances among sampling sites by calculating Haversine distances from latitude and longitude coordinates using the fossil package in R [72]. Finally, we examined how hierarchical variation was distributed among individuals, among transects, and among the high and low elevation sites within each transect using AMOVA [73] as implemented in the R poppr package [74,75]. For this analysis we only used genotype probabilities with relatively high certainty of either the homozygous or the heterozygous genotype (point estimates within 0.1 from 0, 1 or 2), and treated others as missing data. We tested for significance of ϕ statistics with a permutation-based approach using the *randtest* function in the ade4 R package [76].

To test for patterns of parallel locus specific allele frequency shifts across low and high elevation groups, we quantified locus specific *F*_ST_ estimates for all loci for the high and low elevation contrasts at each of the three geographical areas. We considered loci residing above the 97th, 98th and 99th quantiles of the *F*_ST_ distribution for each elevational contrast as outliers potentially residing in genomic regions experiencing directional selection. We evaluated potential parallel differentiation using a permutation-based approach to assess significance of the counts of loci that had extreme *F*_ST_ values in more than one elevational contrast. More specifically, the loci in all three elevational comparisons were permuted and then we counted the number of loci that resided in the upper quantile in multiple elevational comparisons; permutations were conducted 10 000 times to create a null distribution.

## 3. Results

### 3.1. Population genetic structure

One lane of sequencing on the Illumina HiSeq generated 225 302 911 reads. After cleaning contaminants, parsing bar codes, and removing sequences containing pieces of the Illumina adaptors or primers, 185 281 261 reads from 167 individual birds were retained for analyses. *De novo* assembly of a subset of 25 million reads aligned 21 269 517 reads into 313 976 contigs with a coverage depth greater than 10. The consensus sequences of these contigs were retained as a GBS reference onto which we assembled reads from each individual bird. The final reference based assemblies (executed using bwa) placed 143 928 656 reads across all individuals into alignments. Thus, the final alignments contained 77% of the cleaned reads. After removing loci with minor allele frequency < 0.05, we retained genotype estimates for 37 252 loci in the 167 individual birds. We further filtered for SNPs with a minimum coverage depth of 4× and maximum of 15× per locus and a minimum of coverage depth of 3× and a maximum 10× per individual. After this filtering step we retained a final set of genotypes at 18 073 loci in 151 birds that passed our coverage criteria.

**Figure 2.**
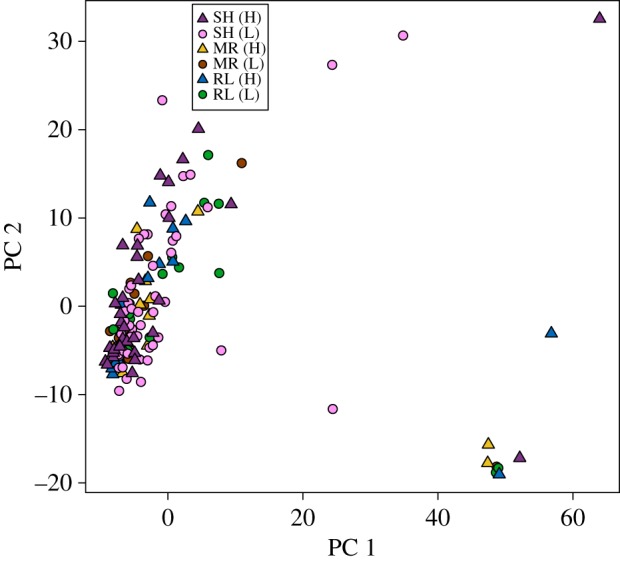
Genetic variation among individual mountain chickadees, as illustrated by the first two principal components from a PCA on the genotype covariance matrix. Individuals from different sampling sites are represented by different colours; high and low elevation sites are labelled in the legend with H and L and plotted as triangles and circles, respectively. MR, Mount Rose; RL, Red Lake; SH, Sagehen.

**Figure 3.**
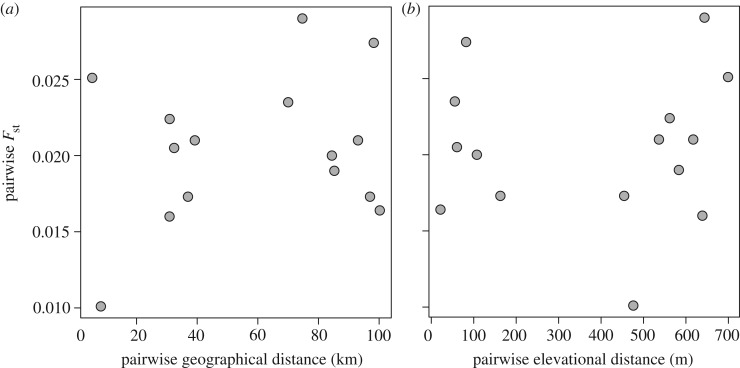
Geographical distance (*a*) and elevational distance (*b*) were not related to pairwise genome-wide *F*_ST_ estimates for each of the six sampling sites. Geography Mantel *R*^2^ = 0.053; *p* = 0.520. Elevation Mantel *R*^2^ = 0.002; *p* = 0.713.

**Figure 4.**
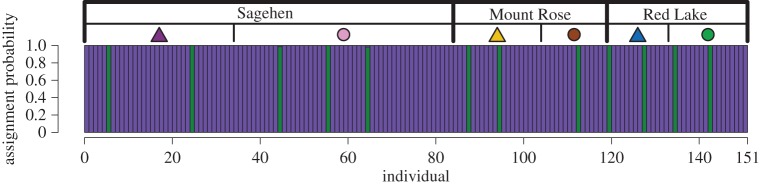
DAPC analyses found support for two genetic clusters of individuals. Individual assignment probabilities for each cluster are plotted, with individuals grouped by sampling locality. Sampling locations are indicated at the top of the plot, and separated by vertical bars. Individuals with high assignment probabilities for each cluster are found in every sampling locality, consistent with a pattern of no population genetic structure.

The first two principal components accounted for 5.77% of the genotypic variation and revealed no evidence that individuals from the six separate sampling sites were more genetically similar to each other than to those from other sites (PERMANOVA *R*^2^ = 0.025; *F*_5,145_ = 0.742; *p* = 0.653) (figure 2). In addition, there was no evidence for overall genetic differentiation among high and low elevation sites (PERMANOVA *R*^2^ = 0.001; *F*_1,149_ = 0.201; *p* = 0.870). Similarly, pairwise genome-wide *F*_ST_ and Nei's *D* estimates for samples of chickadees from different sampling sites were consistent with minimal genetic differentiation (mean *F*_ST_ = 0.020, range: 0.010–0.029). Groups of chickadees from high elevation sampling sites did not consistently exhibit significant genetic differentiation from neighbouring low elevation sites, and the genome-wide *F*_ST_ estimates for most contrasts were not significantly different from zero (table 2). Although some *F*_ST_ estimates were statistically larger than zero, the values of these estimates were very small (table 2). Consistent with the absence of genetic structure among sampling sites, geographical distance was unrelated to genetic distance (Mantel *R*^2^ = 0.053; *p* = 0.520; figure 3*a*) and elevational distance was unrelated to genetic distance (Mantel *R*^2^ = 0.002; *p* = 0.713; figure 3*b*). Hierarchical analysis of genetic variation using AMOVA partitioned nearly all of the variance within samples (99.5%), with minute proportions attributed to variation among transects (0.49%) and across elevations within transects (0.01%). Based on these analyses, there was no evidence for significant genetic structuring among transects (*ϕ* = 0.004; *p* = 0.10) or among different elevations within transects (*ϕ* = −0.001; *p* = 0.59). One PC was retained for the DAPC analysis, which found *K* = 2 as the most likely number of clusters in explaining the data (figure 4). Individuals with high assignment probabilities for each of the two clusters were found at each site and across elevations (figure 4), consistent with a lack of any discernable population structure.

**Table 2.** For each pairwise site comparison, the observed mean *F*_ST_ [69] was compared to a null distribution of *F*_ST_ estimates constructed from 100 permutations of site identity. (Observed mean *F*_ST_ estimates residing outside of the null 95% confidence intervals were deemed significantly larger (*) or smaller (***) than expected by chance. Low (L) and high (H) populations are delineated parenthetically after population abbreviations (MR, Mount Rose; RL, Red Lake; SH, Sagehen).)

site 1	site 2	null mean	null s.d.	95% lower	95% upper	observed mean
MR (L)	RL (H)	0.0279	0.0027	0.0273	0.0284	0.0290*
MR (L)	RL (L)	0.0248	0.0024	0.0243	0.0253	0.0274*
MR (L)	MR (H)	0.0240	0.0019	0.0236	0.0244	0.0251*
MR (L)	SH (H)	0.0201	0.0018	0.0198	0.0205	0.0221*
MR (L)	SH (L)	0.0179	0.0007	0.0178	0.0181	0.0205*
RL (H)	RL (L)	0.0243	0.0021	0.0239	0.0247	0.0224***
RL (H)	MR (H)	0.0247	0.0028	0.0242	0.0253	0.0235***
RL (H)	SH (H)	0.0205	0.0022	0.0201	0.0209	0.0200***
RL (H)	SH (L)	0.0187	0.0011	0.0185	0.0189	0.0190*
RL (L)	MR (H)	0.0215	0.0024	0.0210	0.0219	0.0210
RL (L)	SH (H)	0.0173	0.0015	0.0170	0.0176	0.0173
RL (L)	SH (L)	0.0155	0.0009	0.0153	0.0157	0.0164*
MR (H)	SH (H)	0.0165	0.0013	0.0162	0.0168	0.0173*
MR (H)	SH (L)	0.0146	0.0007	0.0145	0.0148	0.0160*
SH (H)	SH (L)	0.0104	0.0007	0.0103	0.0106	0.0101***

Although there was little evidence for genome-wide genetic differentiation among populations, we examined the distributions of locus specific *F*_ST_ in order to evaluate whether individual loci exhibited elevated differentiation in multiple elevational contrasts, which would be consistent with such loci tagging genomic regions responding to elevation-related selection. There were a small number of loci that were outside the 97th, 98th and 99th quantiles of the genome-wide *F*_ST_ distribution in two elevational contrasts (*N* = 71, 38 and 8, respectively), and few to zero loci that were outside these quantiles in all three elevational contrasts (table 3). For all three *F*_ST_ cut-offs, we observed more loci in the upper *F*_ST_ distributions for two transects than expected from permuted null simulations (table 3). Additionally, there were more loci in the 97th quantile for all three elevational transects (*N* = 2) than expected (table 3). Thus, there is subtle evidence for parallel allele frequency shifts in the same genomic regions. However, all of the loci that did exhibit parallel shifts had *F*_ST_ ≤ 0.3, reflecting relatively weak allele frequency differences even for the very small number of loci that exhibited the most pronounced allele frequency differential.

**Table 3.** Summary data from tests of parallel differentiation for individual loci across multiple transects. (The number of observed loci that were extreme (based on three *F*_ST_ cut-offs) in two or three elevational contrasts was compared to mean expected values based on 10 000 permutations of the loci (95% confidence intervals are listed parenthetically). Observed values greater than expected 95% confidence interval upper bounds are suggestive of parallel differentiation across elevational transects.)

	observed		expected	
*F*_ST_ quantile	2 transects	3 transects	2 transects	3 transects
99th	8	0	5.40 (4.96 to 5.84)	0.015 (−0.01 to 0.04)
98th	38	0	21.27 (20.40 to 22.13)	0.147 (0.07 to 0.22)
97th	71	2	47.33 (46.07 to 48.59)	0.493 (0.36 to 0.63)

## 4. Discussion

Despite evidence of large elevation-related phenotypic divergence at two of our sampling locations (Sagehen Experimental Forest and Mount Rose), we detected little evidence for genetic differentiation between high and low elevation locations or across space for the geographical region we sampled. There was no indication in PCA or DAPC analyses of any population structure (figures 2 and 4), and pairwise *F*_ST_ estimates were small, with many not statistically different from zero. Similarly, geographical and elevation distances were not related to genetic distances (figure 3). Finally, a hierarchical AMOVA revealed no evidence for genetic variation structured by transect or by high and low elevation sites within transects. Mountain chickadees inhabit a continuous gradient of habitat among and within the locations that were sampled, and experience no known geographical barriers to movement. A lack of geographical barriers and the fact that birds can be highly mobile, make the absence of population genetic structure at this geographical scale perhaps unsurprising [43,77,78].

All chickadee species are known to be resident birds that disperse short distances from their natal site and remain fairly sedentary for the rest of their lives [28,45,79]. Preliminary data on natal dispersal and movement in mountain chickadees inhabiting our Sagehen location agree with this previous work, and show that chickadees disperse on average between 0.04 and 2.4 km from their nest-boxes. Despite these sedentary habits, there are few examples of population genetic structure, particularly on small spatial scales, in Paridae. These instances involve limited gene flow between populations as a result of habitat differentiation (e.g. urban park fragmentation in *Parus major* [80] and deciduous versus evergreen oak forests in *Cyanistes caeruleus* [81]). Unlike these examples, our mountain chickadee populations inhabit a continuous gradient of mixed conifer forests. As such, it is possible that even though juveniles in each generation disperse only a short distance, over multiple generations such movements may close the gap between high and low elevations, especially considering that one migrant per generation can abolish any population genetic structure [82,83]. While the conifer species differ as elevation changes, there is no true habitat fragmentation or differentiation, which probably allows enough gene flow to prevent population genetic structure. Given the low marker density our data represent (1 SNP per approximately 83 kb based on an expected genome size of approximately 1.5 Gb (e.g. *P. major* [84])), these SNPs could fail to sample regions of the genome containing variants influencing spatial memory ability between high and low elevation birds.

Instances of loci exhibiting exceptional divergence (outlier loci) across replicated environmental gradients could arise when loci are linked to genomic regions involved in local adaptation (e.g. [3,10,85,86]). Although the elevational gradient that our study populations inhabit could lead to such a pattern, we found little evidence for any of the 18 073 loci we analysed exhibiting parallel patterns of exceptional divergence across high and low elevations. There are several reasons why the genetic variation in our data is unlikely to reflect local adaptation to high and low elevation environments. First, given the low marker density of our data, and expected patterns of linkage disequilibrium in avian genomes (e.g. [87,88]), GBS data may have limited ability to detect genetic regions responding to selection in many cases.

Second, variation in complex quantitative traits often involves large numbers of small effect loci [87,89–95]. Cognition and other behavioural phenotypes that exhibit divergence between low and high elevation chickadees are likely to have a strongly polygenic basis (see review in [94]). Polygenic adaptation resulting from many small effect loci has been notoriously difficult to detect and understand, especially when effective population size is large and linkage disequilibrium decays rapidly [92,95,96]. For example, recent genome-wide studies in birds have failed to detect any variants associated with polygenic phenotypes [97], even when using whole genome resequencing approaches [87]. Understanding the genetic basis of complex behavioural phenotypes such as cognition could require whole genome resequencing studies with very large numbers of individuals. Owing to the immense difficulties and uncertainties involved with quantitative trait mapping [96], it may be that assessing fitness consequences and heritability of cognition and other polygenic behavioural phenotypes may be a more promising approach for detecting evidence of local adaptation.

Previous work on this mountain chickadee system has shown that high and low elevation birds exhibit differences in spatial memory ability, hippocampal morphology, social dominance, novel environment exploration, problem solving and proactive aggression [19,31,39,98,99], as well as significant differences in mate preference [22], and male song structure [23]. In addition, several of these phenotypic differences between high and low elevation chickadees have been documented over multiple years despite large climatic variation among these years [32,54]. If these phenotypes have even moderate heritability, this variation would be consistent with local adaptation, as migrants moving from low to high elevations are likely to experience decreased fitness owing to inferior memory ability [19,32] and assortative mating [22], while migrants moving from high to low elevations are likely to experience decreased fitness owing to their low social dominance status [39].

Adaptation in the presence of gene flow is quite common in many species, including birds [11,14,15,20,81,100–104], however, we do not have definitive evidence that the phenotypic differences between high and low elevation birds are the result of local adaptation rather than phenotypic plasticity. It is likely that some of the variation we see across these numerous phenotypic traits is influenced by phenotypic plasticity resulting from climatic variation in high and low elevation environments. On the other hand, spatial memory ability and hippocampal morphology are moderately to highly heritable, at least in mammals, and therefore are capable of responding to selection [33–38] (also see [94] for in depth review). Assuming there are environment related differences in selection pressures on spatial memory at different elevations (e.g. [19,54]), these differences in selection pressure may result in variation in spatial memory and hippocampal morphology, even in the presence of gene flow.

Overall, our study detected no elevation or geographical related population genetic structure despite numerous phenotypic differences previously documented in mountain chickadees. Future work should now focus on identifying the mechanisms underlying elevation-related phenotypic variation in these birds. In order to understand the role of local adaptation in driving these phenotypic differences, it might be more fruitful to concentrate on measurements of heritability and selection, as opposed to genome-wide association techniques, owing to the well-known difficulties associated with identifying the genetic basis of highly polygenic traits [92,95,96].

## Data accessibility

Genomic data: a directory with .bam files containing sequence alignment data for each individual, and a matrix of genotype probabilities used for population genetic analyses are deposited at Dryad: http://dx.doi.org/10.5061/dryad.686rq [105].
